# Evaluation of Temperature Regulation Efficiency of a Bilayer Coating on Glass with Evaporative and Radiative Cooling for Energy Management

**DOI:** 10.3390/molecules30092042

**Published:** 2025-05-03

**Authors:** Huanying Zhang, Yonghang Yu, Dedong Ji, Chen Zhou, Shengyang Yang

**Affiliations:** 1 Department of Chemistry and Chemical Engineering, Yangzhou University, 180 Siwangting Road, Yangzhou 225002, China; 2 Department of Physical Sciences, University of Central Missouri, Warrensburg, MI 64093, USA

**Keywords:** cooling coating, hydrogel, radiative cooling, evaporative cooling, energy-saving

## Abstract

With the increasing demand for energy-efficient and sustainable building materials, innovative cooling technologies have become a key focus in the construction industry. This study developed a double-layer cooling coating integrating evaporation and radiation mechanisms. The first layer consists of a TiO_2_/PUA radiation layer, where rutile TiO_2_ is incorporated into polyurethane acrylate (PUA) resin to enhance solar reflectivity. The second layer is a P(NVP-co-NMA) hydrogel, which evaporates water at high temperatures and absorbs moisture from the air at low temperatures, eliminating the need for additional water supply systems. The TiO_2_/PUA@P(NVP-co-NMA) coating demonstrates high solar reflectivity and infrared emissivity, effectively reducing indoor temperatures by dissipating heat through water evaporation and radiative cooling. Testing showed a temperature reduction of approximately 7.6 °C in a small house with this coating under simulated conditions. This material demonstrates favorable properties that may make it suitable for applications on building roofs and exterior walls, potentially addressing some limitations of conventional evaporative or radiative cooling systems. Its observed multi-effect cooling performance indicates promise for contributing to energy savings in sustainable building designs.

## 1. Introduction

Global warming has become one of the most pressing challenges for humanity, with increasing temperatures contributing to a host of environmental, economic, and social problems [1,2,3,4,5]. As global temperatures rise, the demand for cooling systems, particularly air conditioners, has soared, leading to excessive energy consumption and exacerbating the effects of global warming [6,7,8]. Buildings, particularly those with large external surface areas, absorb a significant amount of solar radiation, leading to increased internal temperatures [9]. To counter this, energy-intensive cooling systems are employed, contributing to higher energy costs and worsening environmental conditions by increasing the demand for electricity and contributing to greenhouse gas emissions [10]. Reducing the reliance on traditional air-conditioning systems could save significant amounts of energy, reduce costs, and mitigate global warming trends [11].

Two promising strategies to combat this issue are evaporative and radiative cooling, which do not rely on traditional power-consuming systems [12]. Evaporative cooling, in particular, harnesses the natural process of water evaporation to cool the surrounding air without the need for electricity, which, however, often requires water supply systems, which can make it difficult to implement in certain settings [13,14]. Additionally, radiative cooling, which uses the natural ability of materials to emit infrared radiation and cool the surrounding environment, is highly dependent on weather and geographic conditions, limiting its widespread application [15,16]. The cooling efficiency of radiative cooling can also be reduced during cloudy weather or at night when the cooling effect is minimal [17].

To address the limitations of each cooling method, recent advancements have focused on combining the benefits of evaporative and radiative cooling [18,19]. Multi-layered porous materials that integrate these two cooling mechanisms have shown promising results [20,21]. The combination of evaporative and radiative cooling optimizes the cooling performance and endows a system that is more resilient to varying weather conditions and less dependent on external resources like water or electricity [22]. Research has demonstrated that materials that exploit the synergistic effects of both cooling processes can achieve highly effective temperature regulation, contributing to significant energy savings and reduced environmental impacts [23,24].

In the design of advanced cooling materials, the introduction of inorganic fillers has become a common strategy [25]. These fillers are typically chosen for their ability to enhance the optical properties of coatings, particularly in terms of reflecting and scattering solar radiation. Titanium dioxide (TiO_2_), a widely used inorganic filler, is particularly effective due to its high refractive index and low absorption in the near-infrared region [26,27]. TiO_2_, when incorporated into cooling materials, can significantly improve their ability to reflect sunlight and reduce heat absorption, thereby improving their overall cooling efficiency [28,29]. The use of high-refractive-index fillers in cooling materials, such as TiO_2_, plays a crucial role in enhancing the radiative cooling effects by minimizing heat absorption while maximizing the reflection of solar radiation [30,31]. Another important consideration in the development of advanced cooling materials is the use of hydrogels. Hydrogels, networks of polymer chains that can retain large amounts of water, have gained attention for their potential in cooling applications [32]. These hydrogels are particularly effective in enhancing the evaporative cooling effect, as they are capable of absorbing and retaining large volumes of water, which can then evaporate to cool the surrounding environment [33]. Furthermore, their network structure ensures that the water is held within the coating, allowing for prolonged cooling without requiring frequent rehydration [34]. Consequently, the integration of evaporative and radiative cooling systems offers a promising solution for building temperature regulation and energy conservation [35,36].

In this work, we designed a dual-layer cooling coating that integrates evaporative and radiative cooling mechanisms, which has not been explored in this specific combination before. The coatings in this study are fabricated via UV-induced radical polymerization, a standard industrial process for fast-curing polymeric films. The first layer is a TiO_2_/PUA radiation layer, where rutile TiO_2_, known for its high reflectivity in the solar spectrum, is incorporated into a polyurethane acrylate oligomer resin (UA). This resin, synthesized through a polymerization process, enhances the coating’s durability and adhesion to building surfaces while boosting the reflection of sunlight. The second layer consists of a hydrogel copolymer, P(NVP-co-NMA), made from N-vinyl pyrrolidone (NVP) [37] and N-hydroxymethyl acrylamide (NMA), both of which are known for their water retention and polymeric properties [38]. This hydrogel is synthesized using free radical polymerization, which ensures a robust and flexible structure for moisture absorption and retention. The resulting TiO_2_/PUA@P(NVP-co-NMA) coating exhibits high solar reflectivity and infrared emissivity, enabling it to absorb indoor heat, evaporate water into the environment, and radiate infrared electromagnetic waves into space. This study is novel in its specific composition, which combines high-refractive-index TiO_2_ and water-retentive hydrogels, offering a unique solution for temperature regulation in buildings. When applied as a roof coating on a small house with glass as the substrate, the TiO_2_/PUA@P(NVP-co-NMA) coating reduced the temperature by approximately 7.6 °C compared to a plain glass roof, under a room temperature of 25 °C and 0.75 sun intensity. By harnessing both natural cooling processes, this material shows potential as an effective and eco-friendly approach to reducing energy consumption. The combination of these components improves the cooling performance observed in our experiments and contributes to stable operation under the tested environmental conditions.

## 2. Result and Discussion

**Figure 1:**
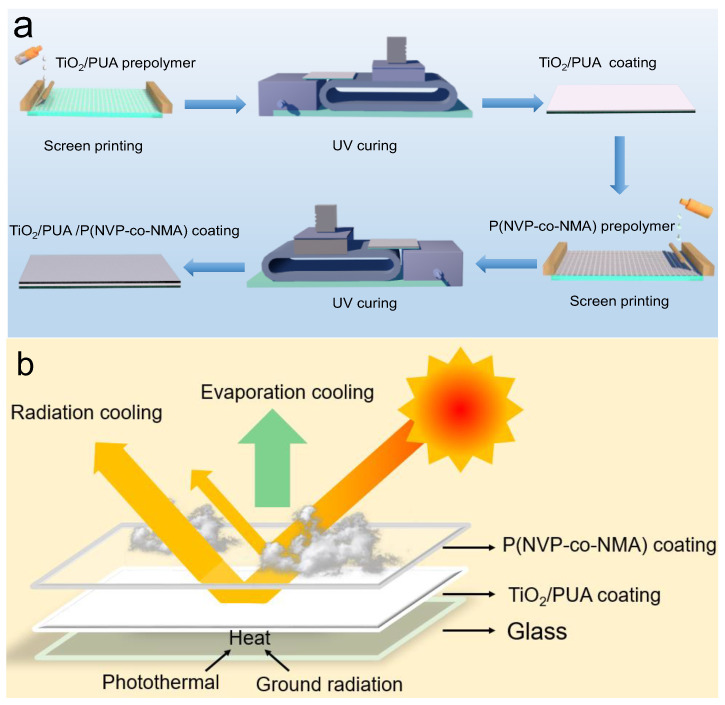
(**a**) Schematic diagram of the synthesis process of the TiO_2_/PUA coating and the TiO_2_/PUA@P(NVP-co-NMA) coating. (**b**) An illustration of the dual processes underlying the temperature regulation mechanism.

An inorganic filler TiO_2_ with high solar reflectivity was incorporated into UA to form a TiO_2_/PUA radiative coating through UV-curable screen printing (Scheme S1). Subsequently, a transparent P(NVP-co-NMA) hydrogel layer (Scheme S2) was constructed atop the TiO_2_/PUA coating using the same preparation process (Figure 1a). This resulted in a TiO_2_/PUA@P(NVP-co-NMA) bilayer cooling coating that integrates evaporative and radiative cooling mechanisms (Figure 1b). The P(NVP-co-NMA) hydrogel layer, being transparent, allows visible light to pass through, which can generate thermal radiation inside the house. However, with the inclusion of the TiO_2_/PUA coating, solar radiation across the entire spectrum is effectively reflected, preventing most sunlight from entering the house. This design ensures efficient cooling under different conditions: when the P(NVP-co-NMA) hydrogel layer is dry or in a water-deficient state, cooling is achieved through radiative cooling alone. Conversely, when the hydrogel contains water, a dual cooling effect is realized through the synergistic processes of evaporative and radiative cooling.

**Figure 2:**
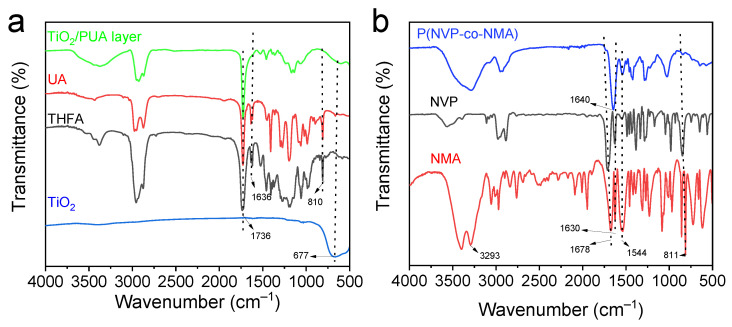
(**a**) FT-IR spectra of TiO_2_/PUA coatings and synthesis of their coating monomers. (**b**) FT-IR spectra of the primary components in the P(NVP-co-NMA) hydrogel formulation, including NVP and NMA and the photocured P(NVP-co-NMA) hydrogel.

To confirm the successful preparation of the coatings, FT-IR analysis was conducted. As shown in Figure 2a, the characteristic absorption peaks of the C=C bond in UA appear at 810 cm^−1^ and 1636 cm^−1^ [39]. In the FT-IR spectra of the TiO_2_/PUA coating, these characteristic peaks at 810 cm^−1^ and 1636 cm^−1^ disappeared, indicating the complete polymerization of polyurethane acrylate under UV irradiation. Additionally, the peaks observed at 1724 cm^−1^ and 1766 cm^−1^ correspond to the C=O stretching vibrations of UA and the TiO_2_/PUA coating, respectively [40]. The characteristic Ti-O absorption peak of TiO_2_ appears at 667 cm^−1^ [41], and the same peak is observed in the TiO_2_/PUA coating, confirming that TiO_2_ was well integrated into the PUA matrix. As shown in Figure 2b, the FT-IR spectra of NMA and NVP display the characteristic C=O absorption peak at 1678 cm^−1^ [42]. The peaks at 1630 cm^−1^ and 811 cm^−1^ correspond to the C=C bond stretching vibrations [43,44], while the peak at 1544 cm^−1^ is attributed to the deformation vibration of the -NHR amide II band in NMA and the pyridinium ion band in NVP, respectively. In the spectrum of P(NVP-co-NMA) (Figure 2b), no absorption peaks corresponding to the C=C double bonds are observed, confirming the successful polymerization and synthesis of the P(NVP-co-NMA) hydrogel.

**Figure 3:**
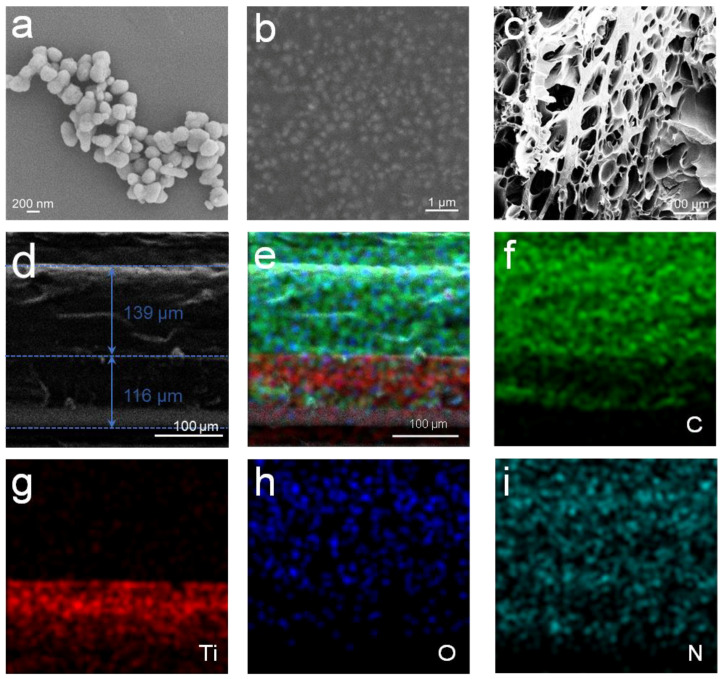
(**a**,**b**) SEM images of (**a**) pure TiO_2_ and (**b**) TiO_2_/PUA coating. (**c**) Cross-sectional image of P(NVP-co-NMA) after freeze-drying. (**d**) SEM image of TiO_2_/PUA@P(NVP-co-NMA) cross section. (**e**) EDS image of TiO_2_/PUA@P(NVP-co-NMA). (**f**–**i**) Distribution of C, Ti, O, and N elements in the cross-section of TiO_2_/PUA@P(NVP-co-NMA).

As observed in the SEM image (Figure 3a), TiO_2_ exhibits a rounded to angular particle profile, and the particles are generally elliptical. As reported in previous studies, the highest light scattering efficiency is achieved when the TiO_2_ particles adopt an elliptical shape [45]. The SEM image confirms that TiO_2_ particles are homogeneously distributed and completely embedded within the PUA resin matrix (Figure 3b). This uniform dispersion indicates excellent compatibility between the inorganic TiO_2_ particles and the polymer matrix, which enhances interfacial adhesion and promotes homogeneous particle dispersion. Figure 3c shows the microscopic morphology of the P(NVP-co-NMA) hydrogel after freeze-drying. Although the highly porous network observed in the freeze-dried hydrogel arises from ice-templating during water sublimation and does not reflect the intrinsic morphology of the hydrated gel, it reveals the underlying polymer matrix architecture. Upon rehydration, this templated network enables rapid water uptake and retention, thereby enhancing the adsorption capacity of hydrogel and its ability to supply water for evaporative cooling. Additionally, the P(NVP-co-NMA) hydrogel maintains good toughness, demonstrating the ability to bend, stretch, and twist, which indicates its structural integrity even under deformation (Figure S1). The P(NVP-co-NMA) hydrogel layer was constructed in situ on top of the TiO_2_/PUA layer following UV curing through screen-printing. To further examine the coating interface, scanning electron microscopy (SEM) was used to observe the adhesion and structural integrity between the TiO_2_/PUA and P(NVP-co-NMA) layers. As shown in the SEM cross-sectional image (Figure 3d), the TiO_2_/PUA coating has a thickness of approximately 116 μm, while the P(NVP-co-NMA) hydrogel layer has a thickness of about 139 μm. The interface between the two layers exhibits a tightly bound and fully dense structure, suggesting strong interfacial adhesion. Energy-dispersive X-ray spectroscopy (EDS) elemental mapping was conducted to analyze the distribution of key elements across the cross-section (Figure 3e). The EDS maps for Si, C, and N clearly distinguish the two layers, revealing a distinctly planar interface that minimizes internal scattering and thus maximizes the intensity of reflected rays, while also indicating strong interlayer bonding. Figure 3f–i further highlight the elemental distributions: C, N, and O are present in both layers, while Ti is exclusively located in the TiO_2_/PUA layer (Figure 3g). The uniform distribution of Ti within the TiO_2_/PUA matrix confirms the homogenous dispersion of TiO_2_ particles within the PUA resin. Overall, the combined SEM and EDS analysis provides clear evidence of the well-defined interface, uniform dispersion of TiO_2_, and the effective construction of the bilayer TiO_2_/PUA@P (NVP-co-NMA) cooling coating. This structural integrity ensures the functional synergy of radiative and evaporative cooling mechanisms.

**Figure 4:**
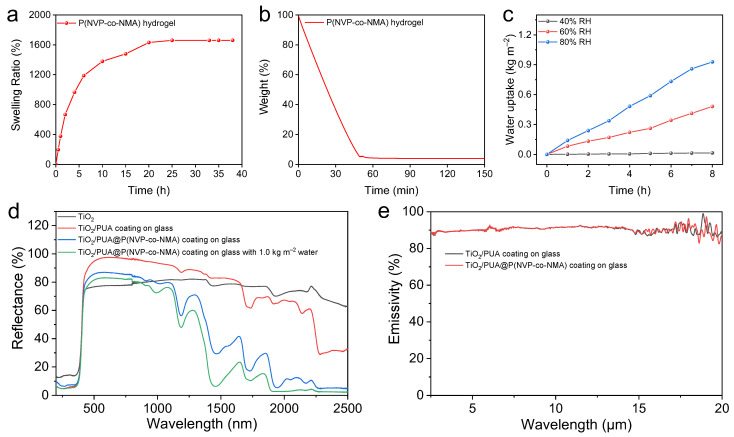
(**a**) Swelling rate of P(NVP-co-NMA) hydrogel with time. (**b**) TGA curves of fully swollen P(NVP-co-NMA) hydrogels at 30 °C for 2.5 h under a nitrogen flow rate of 20 mL min^−1^. (**c**) TiO_2_/PUA@P(NVP-co-NMA) trapping atmospheric water at different humidity. (**d**) The reflectance of pure TiO_2_, glass-based TiO_2_/PUA coating, glass-based TiO_2_/PUA@P(NVP-co-NMA) coating, and glass-based TiO_2_/PUA@P(NVP-co-NMA) coating containing 1.0 kg m^−2^ water in the region of 200–2500 nm. (**e**) The emissivity of the glass-based TiO_2_/PUA coating in the wavelength range of 2.5–20 μm and that of the glass-based TiO_2_/PUA@P(NVP-co-NMA) coating.

The water absorption and storage capacity of the hydrogel is a key factor in achieving evaporative cooling, so the swelling ratio test was conducted to explore the water absorption capacity of hydrogel. The dried hydrogel was directly put into water, taken out at regular intervals to remove the water on the surface, and then weighed. Figure 4a shows that after the hydrogel was put into the water to absorb water for 25 h, its weight no longer changed, and its swelling ratio was as high as 1660%. Meanwhile, the TGA test was carried out on the fully saturated hydrogel, and the temperature was kept at 30 °C for 150 min. Its water content can reach about 96% (Figure 4b). The P(NVP-co-NMA) hydrogel reveals excellent water storage and absorption capacity. The excellent water absorption and storage properties mainly originated from the three-dimensional spatial network structure constructed inside, which could effectively absorb and store a large amount of water inside the hydrogel (Figure 3c). To assess the performance of the P(NVP-co-NMA) hydrogel after water absorption and drying, we conducted multiple drying cycles at 60 °C on the hydrogel, which initially contained 96% water. After 10 cycles, no significant structural changes or yellowing were observed (Figure S2), indicating that the hydrogel is stable even under conditions of high temperature and drying. This further suggests that the hydrogel can store water for a long time and maintain its performance under various environmental conditions, including high temperatures and dry environments. The ability of the TiO_2_/PUA@P(NVP-co-NMA) coating to capture atmospheric water vapor was also explored. Through Figure 4c, it can be observed that the TiO_2_/PUA@P(NVP-co-NMA) coating can collect airborne water when the relative humidity in the air exceeds 40%. When the TiO_2_/PUA@P(NVP-co-NMA) coating is exposed to 80% relative humidity for up to 8 h, it can absorb airborne water of 0.93 kg m^−2^. This indicates that the TiO_2_/PUA@P(NVP-co-NMA) coating does not require a water supply attachment for evaporative cooling. Therefore, the TiO_2_/PUA@P(NVP-co-NMA) coating with a water content of 1.0 kg m^−2^ was used for subsequent experiments.

Since the visible and near-infrared portions account for 95% of the total sunlight, the cooling materials were designed to enhance the reflectivity in these regions [46]. The reflectivity of pure TiO_2_, glass-based TiO_2_/PUA coating, glass-based TiO_2_/PUA@P(NVP-co-NMA) coating, and glass-based TiO_2_/PUA@P(NVP-co-NMA) coating containing 1.0 kg m^−2^ of water were investigated for the region of 200–2500 nm. It can be observed from Figure 4d that TiO_2_ exhibits high reflectivity in both visible and near-infrared regions. The reflectivity of the TiO_2_/PUA coating in these regions is higher than that of pure TiO_2_, which is mainly because the TiO_2_ particles may form an aggregated structure in the PUA film, which increases the scattering of light and thus improves the reflectivity of TiO_2_/PUA. However, the reflectivity of TiO_2_/PUA@P(NVP-co-NMA) coatings, as well as TiO_2_/PUA@P(NVP-co-NMA) coatings containing water, was similar to that of pure TiO_2_ in the visible region but lower in the near-infrared region. This is because the TiO_2_/PUA coating is covered with a transparent P(NVP-co-NMA) hydrogel layer, as well as the fact that water absorbs some of the light in the near-infrared region, resulting in a decrease in reflectivity. However, despite the decreased reflectivity, the heat absorbed in these regions can be expelled to the outside of the building through the evaporation of water, achieving an effective cooling effect. Emissivity can be used to reflect the ability of a material to release surface heat. So, the emissivity of the TiO_2_/PUA coating and the TiO_2_/PUA@P(NVP-co-NMA) coating in the range of 2.5–20 μm was also explored. Through Figure 4e, it can be found that the TiO_2_/PUA coating and TiO_2_/PUA@P(NVP-co-NMA) coating have high emissivity, i.e., around 90%. There is no doubt that increasing the emissivity is favorable to the exothermic property, so the addition of TiO_2_ can improve the cooling performance of the composite film.

To verify that the coatings have good adhesion, the TiO_2_/PUA coatings and TiO_2_/PUA@P(NVP-co-NMA) coatings were tested for adhesion, respectively. After immersing the TiO_2_/PUA-coated samples in boiling water for 24 h, no peeling or bubbling was observed on the surface (Figure S3a). Subsequently, the coating was subjected to a scratch test with dozens of meshes, followed by 10 tensile tests using adhesive tape (3M, 610), and no peeling of the coating was observed (Figure S3b,c), confirming the excellent water resistance of the TiO_2_/PUA layer. The TiO_2_/PUA coatings have excellent adhesion. This excellent adhesion is attributed to the inclusion of both urethane acrylate (UA) and GPTMS coupling agents in the formulation [47]. The P(NVP-co-NMA) hydrogel coating on the glass remained intact with the aid of the TiO_2_/PUA coating adhesive layer after 100 tape peeling tests in sequence, a 100 frames of scratching, and 10 incidences of tape peeling (Figure S3d–f).

**Figure 5:**
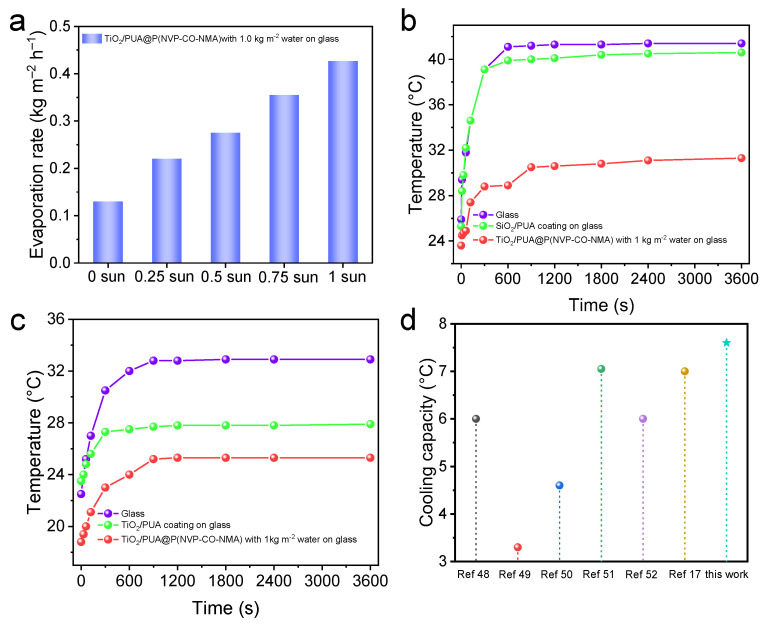
(**a**) Evaporation rate of TiO_2_/PUA@P(NVP-co-NMA) coating on glass containing 1 kg m^−2^ water at a temperature of 25 °C in different light. (**b**) Surface temperature change of glass, the TiO_2_/PUA coating on glass, and the TiO_2_/PUA@P(NVP-co-NMA) coating on glass containing 1.0 kg m^−2^ water at a temperature of 25 °C for 0.75 solar irradiance intensity. (**c**) Temperature variation with time in a glass-covered polystyrene foam box with different coatings at 25 °C, a relative humidity of 33%, and a light intensity of 0.75 suns. (**d**) Comparison of steady-state cooling temperature reductions for the present work and literature-reported systems.

The evaporation rate refers to the rate at which a liquid surface transitions from a liquid to a gas state per unit of time. Water evaporation requires heat absorption, and for building cooling, the evaporation rate directly impacts the effectiveness of the cooling process. The higher the evaporation rate, the more heat can be dissipated simultaneously, leading to a stronger cooling effect. Here, the evaporation rate of TiO_2_/PUA@P(NVP-co-NMA) coatings, containing 1.0 kg m^−2^ of water, was investigated under varying light intensities. As shown in Figure 5a, the evaporation rate of the TiO_2_/PUA@P(NVP-co-NMA) coatings on glass increases with light intensity. At 25 °C, the evaporation rate of the TiO_2_/PUA@P(NVP-co-NMA) coating with 1.0 kg m^−2^ of water is 0.13 kg m^−2^ h^−1^ in the absence of light. Under the same temperature conditions, at a light intensity of 0.75 suns, the evaporation rate increases to 0.35 kg m^−2^ h^−1^, demonstrating the coating’s excellent water evaporation capability. To further evaluate the cooling effect, the surface temperatures of the samples were measured using an infrared thermography camera at 25 °C and 0.75 suns (Figure 5b). The surface temperatures of glass (Figure S4), the glass-based TiO_2_/PUA coating (Figure S5), and glass-based coatings containing 1.0 kg m^−2^ of TiO_2_/PUA@P(NVP-co-NMA) with water (Figure S6) all increased rapidly within the first 15 min, reaching dynamic equilibrium after that period. The surface temperature of the TiO_2_/PUA@P(NVP-co-NMA) coating containing water was approximately 10 °C lower than that of the glass surface, whereas the TiO_2_/PUA coating showed only a 1 °C reduction. This confirms the superior cooling effect of the TiO_2_/PUA@P(NVP-co-NMA) coating containing 1.0 kg m^−2^ of water.

To substantiate the efficacy of the TiO_2_/PUA@P(NVP-co-NMA) coating in alleviating the greenhouse effect, three types of substrates were tested: glass, TiO_2_/PUA-coated glass, and TiO_2_/PUA@P(NVP-co-NMA)-coated glass with 1.0 kg m^−2^ of water. These coatings were installed on three identical expanded polystyrene (EPS) boxes, which served as model rooftops. A xenon lamp equipped with an AM1.5 filter was used to simulate solar irradiation. To accurately replicate the light intensity encountered by outdoor buildings, the intensity was calibrated to 0.75 suns for the building cooling experiment, with thermocouples monitoring temperature variations in real time over one hour. As illustrated in Figure 5c, under illumination, the temperatures within all three boxes initially rose and then plateaued. The temperature inside the box with the TiO_2_/PUA coating was approximately 5 °C lower than that inside the box with plain glass, primarily due to the radiative cooling effect. In contrast, the temperature inside the box with the TiO_2_/PUA@P(NVP-co-NMA) coating containing 1.0 kg m^−2^ of water was approximately 7.6 °C lower than that inside the glass box, benefiting from the synergistic effects of both radiative cooling and evaporative cooling. This suggests that evaporative cooling contributed to an additional temperature reduction of approximately 2.6 °C. These findings demonstrate that the combined effects of evaporative and radiative cooling can achieve a more significant cooling effect. Furthermore, comparisons with other relevant studies on radiative cooling indicate that this study achieved superior cooling performance compared to standalone radiative cooling solutions (Figure 5d) [17,48,49,50,51,52]. This further confirms that the synergistic interaction of radiative cooling and evaporative cooling in buildings can lead to more substantial energy savings.

**Figure 6:**
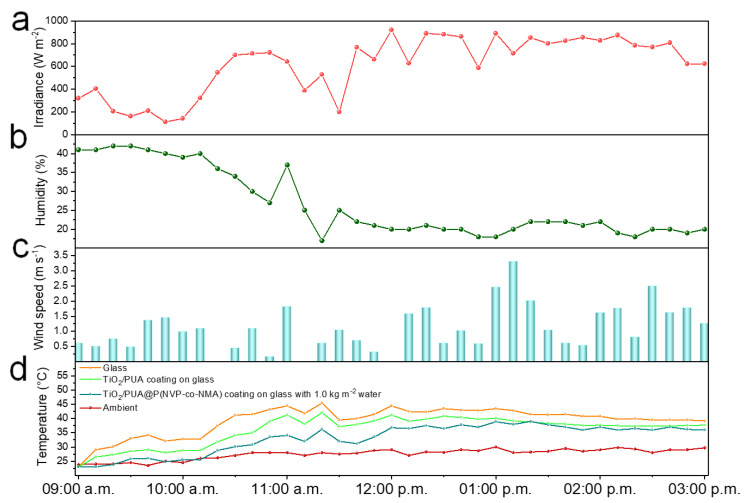
Real-time outdoor environmental parameters, including (**a**) solar irradiance, (**b**) wind speed, and (**c**) humidity. (**d**) The real-time temperatures of the surrounding environment, EPS boxes equipped with glass, TiO_2_/PUA-coated glass, and TiO_2_/PUA@P(NVP-co-NMA) coating with 1.0 kg m^−2^ water on glass were recorded.

To evaluate the real-world cooling performance, outdoor experiments were conducted in practice. Glass, TiO_2_/PUA-coated glass, and TiO_2_/PUA@P(NVP-co-NMA)-coated glass containing 1.0 kg m^−2^ of water were placed at a 45° angle on three identical trapezoidal EPS boxes. Real-time monitoring of outdoor conditions was carried out, including solar irradiance (Figure 6a), wind speed (Figure 6b), and ambient humidity (Figure 6c). As shown in Figure 6d, due to the greenhouse effect, the temperature inside the glass box was significantly higher than the ambient temperature, with an average increase of 12.1 °C. In contrast, the average temperature inside the TiO_2_/PUA@P(NVP-co-NMA) box containing 1.0 kg m^−2^ of water was only about 5.7 °C higher than the ambient temperature, which was 6.4 °C lower than the average temperature inside the glass box. The average temperature inside the TiO_2_/PUA-coated box was 3.3 °C lower than that inside the glass box. These results closely align with the indoor experiment findings. Additionally, as the water content of the TiO_2_/PUA@P(NVP-co-NMA) coating decreased, the temperature difference between the glass box and the TiO_2_/PUA@P(NVP-co-NMA) box gradually narrowed. These outdoor results confirm the practical feasibility of the TiO_2_/PUA@P(NVP-co-NMA) coating containing 1.0 kg m^−2^ of water in mitigating the greenhouse effect.

## 3. Experimental Section

### 3.1. Materials

Titanium dioxide (TiO_2_, 97%) was supplied by DuPont Chemical Reagent Co., Ltd. (Shanghai, China). Urethane acrylate (UA, 100%), a difunctional prepolymer, was obtained from Guangzhou Sanwang Chemical Reagent Co., Ltd. (Guangzhou, China), with trade name SW3670. Tetrahydrofurfuryl acrylate (THFA, 98%), 3-aminopropyltrimethoxysilane (APTS, 97%), and 3-glycidoxypropyltrimethoxysilane (GPTMS, 98%) were purchased from Nanjing Reagent Co., Ltd. (Nanjing, China). Ethyl 2,4,6-trimethylbenzamido phenylphosphonate (TPO-L, 99.8%), Antioxidant-168 (99%) and Antioxidant-1010 (99%) were provided by BASF Chemical Reagent Co., Ltd. (Shanghai, China). *N*-vinylpyrrolidone (NVP, 99%) and N-hydroxymethacrylamide (NMA, 98%) were purchased from McLean Biochemical Technology Co., Ltd. (Shanghai, China). *N*,*N*-methylenebisacrylamide (MBAA, CP) was supplied by Sinopharm Chemical Reagent Co., Ltd. (Shanghai, China).

### 3.2. Preparation of TiO_2_/PUA Coating

The TiO_2_/PUA coating was prepared via the ultraviolet (UV) light-curing screen-printing method. THFA (3 g, 29.4 mmol) and UA (2.6 g) were placed in a brown reagent bottle and mixed homogeneously by sonication. GPTMS (0.5 g, 2.11 mmol) and APTS (0.1 g, 0.56 mmol) were then added to the solution under stirring. Next, 3.5 g TiO_2_ (35 wt%) was introduced into the mixture, and mechanical stirring was conducted for 30 min. Ultrasonication was applied for an additional 15 min to ensure the homogeneous dispersion of the TiO_2_ in the solution. Subsequently, 0.3 g of photoinitiator TPO-L (0.95 mmol), 0.03 g of antioxidant-168 (46.4 µmol), and 0.015 g of antioxidant-1010 (12.74 µmol) were added and uniformly dispersed by ultrasonication to obtain the polymerization solution, which can improve the yellowing resistance of the coating [53]. After screen printing, the polymerization solution was evenly spread on the glass substrate, and a TiO_2_/PUA coating was obtained after 10 min of UV irradiation (0.2 suns).

### 3.3. Fabrication of TiO_2_/PUA@P(NVP-co-NMA) Coating

TiO_2_/PUA@P(NVP-co-NMA) coatings were obtained by the in situ deposition of P(NVP-co-NMA) hydrogel coatings on TiO_2_/PUA layers using the same printing process as that used for the preparation of TiO_2_/PUA coatings. The specific operation was as follows: 4.27 g of NVP (38.4 mmol) and 1.67 g of NMA (16.5 mmol) were added to a beaker containing 4 g of ultrapure water, and after ultrasonically dispersing homogeneously, 5.94 mg of the crosslinking agent MBAA (38.4 µmol) and 17.82 mg of the photoinitiator TPO-L were added to the above solution, and after ultrasonically dispersing homogeneously, a solution to be polymerized was obtained. The solution to be polymerized was also evenly distributed on the TiO_2_/PUA coating by screen printing, and the TiO_2_/PUA@P(NVP-co-NMA) bilayer coating was obtained by UV curing.

### 3.4. P(NVP-co-NMA) Hydrogel Swelling Property Test

To evaluate the swelling rate of P(NVP-co-NMA) hydrogels, the hydrogels were prepared following the same formulation as described in the experimental section. The hydrogel precursor solution was placed in a rectangular container and subjected to light curing to form a dry hydrogel. Due to the photothermal effect, the surrounding humidity was approximately 25–30%. The dry hydrogels were repeatedly immersed in water, then removed to eliminate surface water, and weighed. This process was repeated until the measured mass remained constant over three consecutive measurements, conducted at room temperature (25 °C) and 50% relative humidity. The swelling ratio (SR) was calculated as the weight of water absorbed divided by the weight of the original hydrogel, using the following formula:

$$\mathrm{SR}(\%)=\frac{W_{t}-W_{0}}{W_{0}}$$

where W_0_ and W_t_ are the original mass of the hydrogel and the mass of water absorbed by the hydrogel at moment t, respectively.

### 3.5. TiO_2_/PUA@P(NVP-co-NMA) Coating Water Collection Test from Air

To measure the ability of the TiO_2_/PUA@P(NVP-co-NMA) coating to trap water in the air, the TiO_2_/PUA@P(NVP-co-NMA)-coated glass was placed in a sealed EPS foam box (50 × 50 × 50 cm). A small commercial humidifier was used to regulate the humidity level inside the box, while a hygrometer was used to monitor the humidity. The humidity levels used in the experiments were 40%, 60%, and 80%. Once the humidity reached the desired level, the humidifier was turned off. If the humidity dropped below the expected value, the humidifier was turned back on.

### 3.6. Coating Adhesion Test

Boiling test: We put the samples into 100 °C water for 24 h and take them out to observe whether the coating comes off. Tape Peel Test: Adhesion between TiO_2_@PUA coating and glass and TiO_2_/PUA@P(NVP-co-NMA) coating and glass was tested using adhesive tape (3M, 610). A 1 kg roller was rolled over the tape twice to ensure that the tape firmly adhered to the coatings, and then the tape was quickly peeled off the coating. We repeat the procedure 10 times and observe whether the coating comes off the glass. Scratch test: We scratch the coating with a scratching tool and peel the tape off 100 times to see if the coating comes off the glass.

### 3.7. Cooling Performance Test

The cooling performance of glass, coated glass, and water-containing coated glass was experimentally investigated at an ambient condition of 25 °C and 33% humidity. A xenon lamp with an AM1.5 filter was used to simulate sunlight, an optical power densitometer (CEL-NP2000) was used to measure the light intensity, and an infrared thermal imager (testo 865) was used to measure the temperature of the sample surface. For cooling performance tests, the samples were placed on a floor covered with expanded polystyrene (thermal conductivity of 0.035 W m^−1^ K^−1^, density of 30 kg m^−3^, and thickness of 3 cm). Under these conditions, cooling experiments were conducted at 0.75 solar intensities.

### 3.8. Simulated House Cooling Experiment

A xenon lamp (CEL-HKF300) with an AM1.5 filter was used to simulate solar radiation by adjusting the light intensity to 0.75 suns (750 W m^−2^). To compare the cooling effects of glass and coated glass, three identical model houses were constructed. The floor and walls of these houses were made of expanded polystyrene foam, with a thermal conductivity of 0.024 W m^−1^ K^−1^, a density of 30 kg m^−3^, and a thickness of 0.02 m. Each house had a roof area of 196 cm^2^ made of glass, TiO_2_@PUA-coated glass, and TiO_2_/PUA@P(NVP-co-NMA)-coated glass containing 1.0 kg m^−2^ of water, respectively. A thermocouple was placed in the center of each house to measure the air temperature inside. A staple-free adhesive was used to ensure good thermal contact between the glazing and the roof. The solar simulator was aimed vertically into the houses.

### 3.9. Outdoor Experiment

To verify the practicality of the cooling performance of the coating, field tests were conducted outdoors. The perimeter and bottom of the three model houses were made of polystyrene foam to reduce heat transfer. The tops were covered with glass, TiO_2_/PUA-coated glass, and TiO_2_/PUA@P(NVP-co-NMA)-coated glass containing 1.0 kg m^−2^ of water, respectively. The three model houses were placed on a one-meter-high platform to reduce radiation from the ground. An optical power densitometer was used to measure the solar irradiance. Type K thermocouples were used to measure the temperature inside the model houses and the environment, and an anemometer with a hygrometer was used to measure the outside wind speed and the ambient humidity.

### 3.10. Characterization

Scanning electron microscopy (SEM, GeminiSEM 300, Carl Zeiss, Germany) was used to observe the morphology and structure of the hydrogels after freeze-drying and the thickness and elemental distribution of the coatings. Fourier transform infrared spectroscopy (FT-IR, Antaris II, Thermo Fisher Scientific Inc., Waltham, MA, USA) characterized the functional groups of the PUA coatings and hydrogels and their monomers. The reflectance and absorbance of the samples at 200–2500 nm were determined using a UV–visible near-infrared absorption spectrometer (Cary 5000, Varian Inc., Palo Alto, CA, USA), and the infrared emissivity in the wavelength range of 2.5–20 μm was measured by Fourier transform infrared spectroscopy with solid attachment (FT-IR, Antaris II, Thermo Fisher Scientific Inc., Waltham, MA, USA). Before conducting reflectance, absorbance, and emission tests, powder samples were compacted using a tablet press at a pressure of 15 MPa. Temperature variations within the small houses during the experiments were measured by thermocouples. Light intensity was measured by an optical power densitometer.

## 4. Conclusions

In summary, we have designed a dual-layer cooling coating that integrates evaporative and radiative cooling mechanisms to alleviate building electricity demands and mitigate the greenhouse effect. The radiative layer, composed of TiO_2_ incorporated into polyurethane acrylate (PUA) resin via screen printing and UV curing, demonstrates strong adhesion and high solar reflectance. A transparent P(NVP-co-NMA) hydrogel evaporative layer was applied, enabling water evaporation in an open system and passive water regeneration from the air at moderate humidity or above without requiring additional water supply. The resulting TiO_2_/PUA@P(NVP-co-NMA) coating, containing 1.0 kg m^−2^ of water, exhibits high visible light reflectance and infrared emissivity, achieving a temperature reduction of approximately 7.6 °C under 0.75 solar irradiance compared to conventional glass roofs. While the study demonstrates the potential of multi-effect cooling technologies for building applications, further investigation is needed to evaluate the influence of layer thickness and TiO_2_ content on the coating’s overall cooling performance, providing strong support for the development of next-generation energy-efficient building materials.

## Data Availability

Data are available on request to the corresponding author.

## Supplementary Materials

The following supporting information can be downloaded at https://www.mdpi.com/article/10.3390/molecules30092042/s1: Scheme S1: Reaction in TiO_2_/PUA coating synthesis; Scheme S2: Reaction in the synthesis of P(NVP-co-NMA); Figure S1: Original, bent, stretched, and twisted P(NVP-co-NMA) hydrogel; Figure S2: Original P(NVP-co-NMA) hydrogel and P(NVP-co-NMA) hydrogel after 10 absorption-drying cycles; Figure S3: TiO_2_/PUA-coated glass after 24 h in boiling water, after 100 peel-offs of adhesive tape, after 3M 610 tape scratch test with 10 peel-offs, and TiO_2_/PUA@P(NVP-co-NMA)-coated glass after 100 peel-offs and 10 scratch peel tests; Figure S4: Infrared thermal images of the glass surface under light irradiation; Figure S5: Infrared thermal images of TiO_2_/PUA-coated surface on glass under light irradiation; Figure S6: Infrared thermal images of TiO_2_/PUA@P(NVP-co-NMA) surface containing 1 kg m^−2^ water on glass under light irradiation.
