# Supplementary material for: Evaluation of Temperature Regulation Efficiency of a Bilayer Coating on Glass with Evaporative and Radiative Cooling for Energy Management
Source: Molecules. 2025 May 3;30(9):2042. doi: 10.3390/molecules30092042 (PMC12073255; doi:10.3390/molecules30092042)
Supplement: Supplementary file 1 [file molecules-30-02042-s001.zip › molecules-3421113-supplementary.pdf]

# Supplementary Materials

## Evaluation of Temperature Regulation Efficiency of a Bilayer Coating on Glass with Evaporative and Radiative Cooling for Energy Management

Huanying Zhang,[1] Yonghang Yu,[1] Dedong Ji,[1] Chen Zhou,[2,*] Shengyang Yang,[1,*]

[1] Department of Chemistry and Chemical Engineering, Yangzhou University, 180 Siwangting Road, Yangzhou 225002, China

[2] Department of Physical Sciences, University of Central Missouri, Warrensburg, MI 64093, USA

* Correspondence: zhou@ucmo.edu (C.Z.); syyang@yzu.edu.cn (S.Y.)

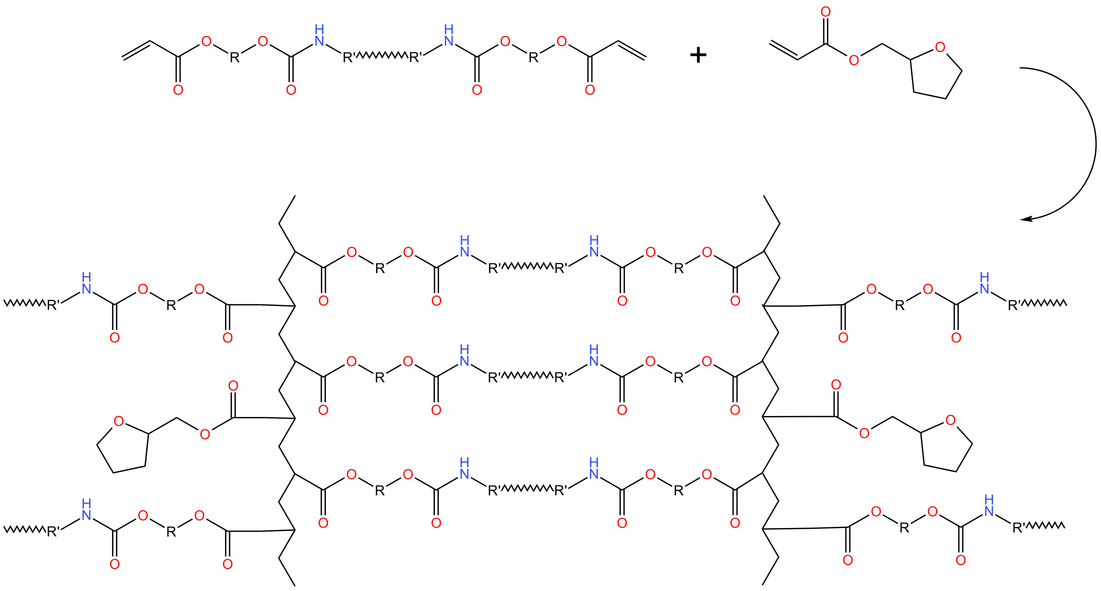

**Scheme S1.** The main reaction in the synthesis of $TiO_2$/PUA coating.

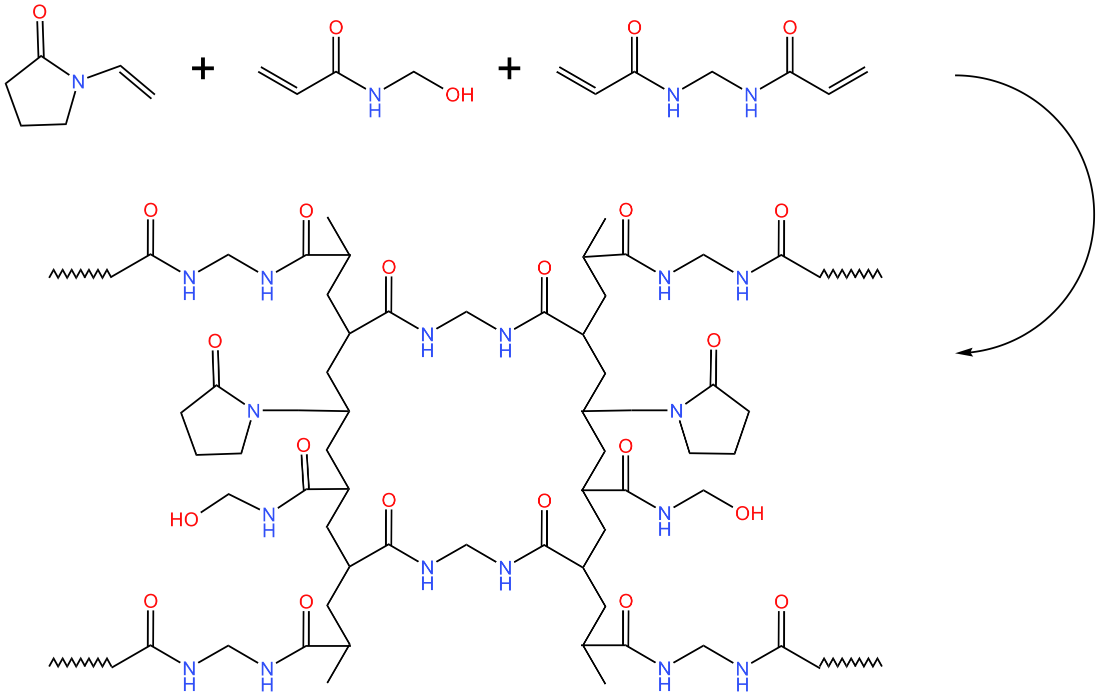

**Scheme S2.** The main reaction in the synthesis of P(NVP-co-NMA) hydrogel layer.

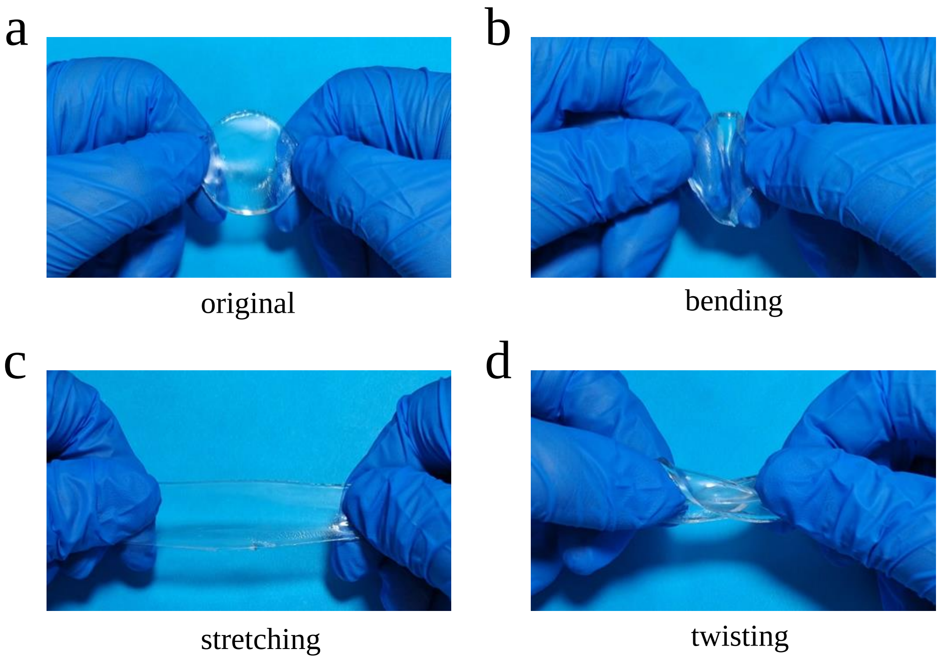


**Figure S1.** (a-d) Photographs of (a) original P(NVP-co-NMA) hydrogel, (b) bent P(NVP-co-NMA) hydrogel, (c) stretched P(NVP-co-NMA) hydrogel, and (d) twisted P(NVP-co-NMA) hydrogel.

a b

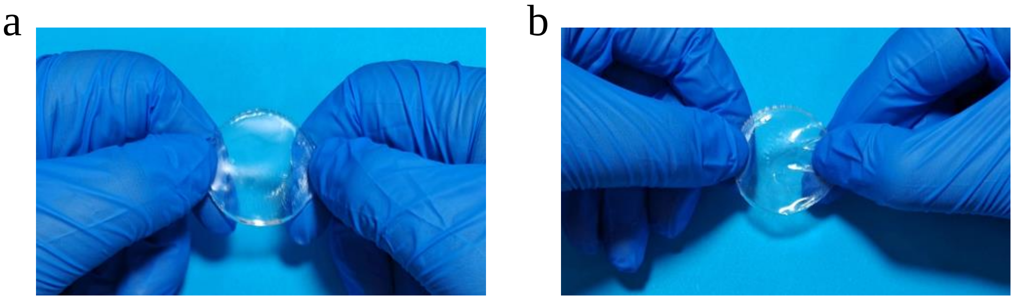

**Figure S2.** (a) Photograph of the original P(NVP-co-NMA) hydrogel. (b) Photograph of the dried P(NVP-co-NMA) hydrogel after undergoing water absorption and drying for 10 cycles.

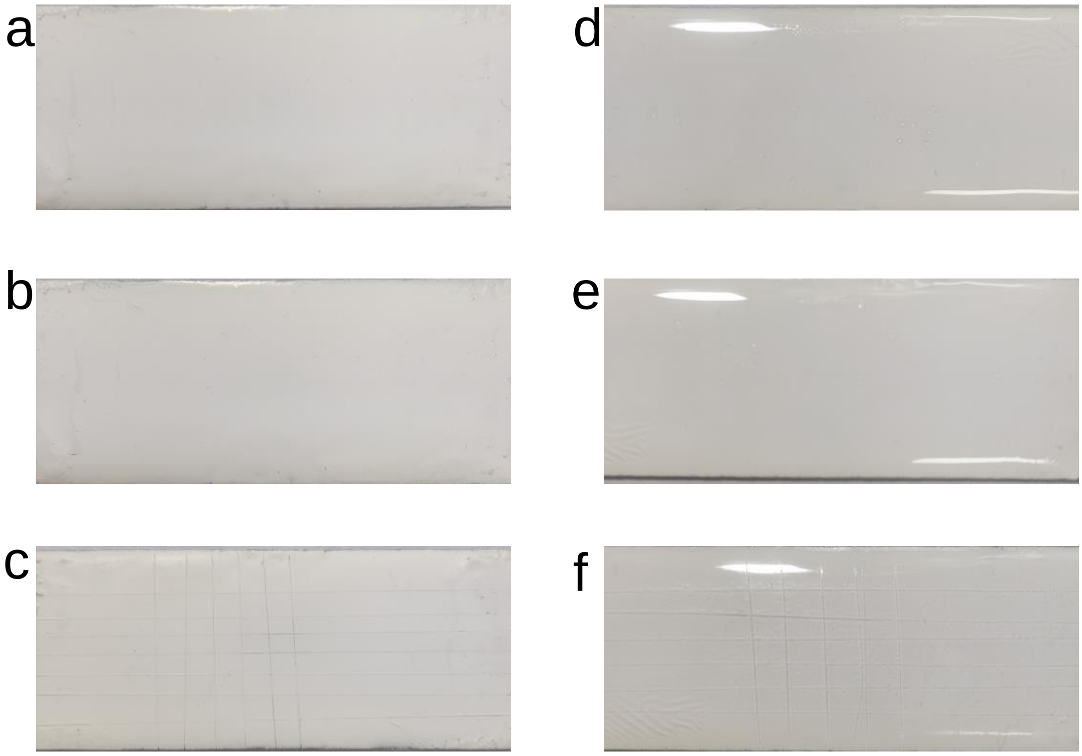


**Figure S3.** (a) Photograph of $TiO_2$/PUA coated glass after 24 h in boiling water. (b) Photograph of $TiO_2$/PUA coated glass after 100 peel-offs of adhesive tape. (c) Photograph of $TiO_2$/PUA coated glass after 3M 610 tape scratch test with 10 tape peel-offs. (d) Photograph of the $TiO_2$/PUA@P(NVP-co-NMA) coated glass. (e) Photograph of $TiO_2$/PUA@P(NVP-co-NMA) coated glass after 100 peel-off tests of adhesive tape. (f) Photo of $TiO_2$/PUA@P(NVP-co-NMA) coated glass after 10 times scratch peel test.

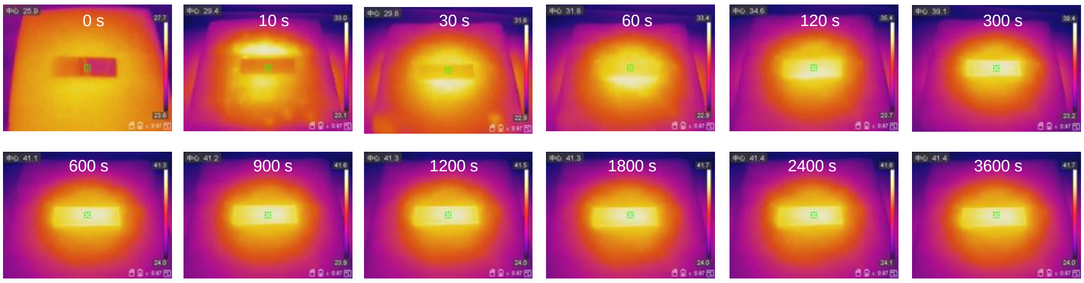


**Figure S4.** Infrared thermal images of the glass surface at 25 °C.

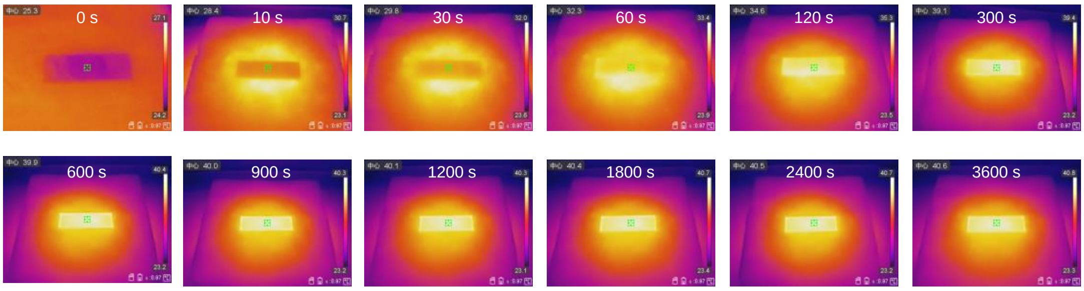


**Figure S5.** Infrared thermal images of $TiO_2$/PUA coated surface on glass at 25 °C.

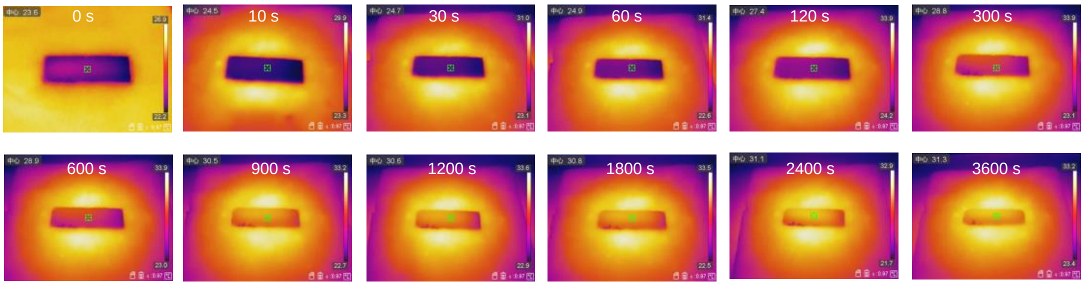


**Figure S6.** Infrared thermal images of $TiO_2$/PUA@P(NVP-co-NMA) surface containing 1 kg $m^{-2}$ water on glass at 25 °C.
